# Complex Patterns of Chromosome 11 Aberrations in Myeloid Malignancies Target *CBL, MLL, DDB1* and *LMO2*

**DOI:** 10.1371/journal.pone.0077819

**Published:** 2013-10-16

**Authors:** Thorsten Klampfl, Jelena D. Milosevic, Ana Puda, Andreas Schönegger, Klaudia Bagienski, Tiina Berg, Ashot S. Harutyunyan, Bettina Gisslinger, Elisa Rumi, Luca Malcovati, Daniela Pietra, Chiara Elena, Matteo Giovanni Della Porta, Lisa Pieri, Paola Guglielmelli, Christoph Bock, Michael Doubek, Dana Dvorakova, Nada Suvajdzic, Dragica Tomin, Natasa Tosic, Zdenek Racil, Michael Steurer, Sonja Pavlovic, Alessandro M. Vannucchi, Mario Cazzola, Heinz Gisslinger, Robert Kralovics

**Affiliations:** 1 CeMM Research Center for Molecular Medicine of the Austrian Academy of Sciences, Vienna, Austria; 2 Division of Hematology and Blood Coagulation, Department of Internal Medicine I, Medical University of Vienna, Vienna, Austria; 3 Department of Hematology Oncology, Fondazione IRCCS Policlinico San Matteo, Pavia, Italy; 4 Department of Molecular Medicine, University of Pavia, Pavia, Italy; 5 Section of Hematology, University of Florence, Florence, Italy; 6 Department of Internal Medicine Hematology and Oncology, University Hospital Brno, Masaryk University Brno, Brno, Czech Republic, Czech Republic; 7 CEITEC - Central European Institute of Technology, Masaryk University Brno, Brno, Czech Republic; 8 Clinic of Hematology, Clinical Center of Serbia, University of Belgrade, School of Medicine, Belgrade, Serbia; 9 Institute of Molecular Genetics and Genetic Engineering, University of Belgrade, Belgrade, Serbia; 10 Division of Hematology and Oncology, Innsbruck University Hospital, Innsbruck, Austria; Ohio State University Medical Center, United States of America

## Abstract

Exome sequencing of primary tumors identifies complex somatic mutation patterns. Assignment of relevance of individual somatic mutations is difficult and poses the next challenge for interpretation of next generation sequencing data. Here we present an approach how exome sequencing in combination with SNP microarray data may identify targets of chromosomal aberrations in myeloid malignancies. The rationale of this approach is that hotspots of chromosomal aberrations might also harbor point mutations in the target genes of deletions, gains or uniparental disomies (UPDs). Chromosome 11 is a frequent target of lesions in myeloid malignancies. Therefore, we studied chromosome 11 in a total of 813 samples from 773 individual patients with different myeloid malignancies by SNP microarrays and complemented the data with exome sequencing in selected cases exhibiting chromosome 11 defects. We found gains, losses and UPDs of chromosome 11 in 52 of the 813 samples (6.4%). Chromosome 11q UPDs frequently associated with mutations of *CBL*. In one patient the 11qUPD amplified somatic mutations in both *CBL* and the DNA repair gene *DDB1*. A duplication within *MLL* exon 3 was detected in another patient with 11qUPD. We identified several common deleted regions (CDR) on chromosome 11. One of the CDRs associated with *de novo* acute myeloid leukemia (P=0.013). One patient with a deletion at the *LMO2* locus harbored an additional point mutation on the other allele indicating that *LMO2* might be a tumor suppressor frequently targeted by 11p deletions. Our chromosome-centered analysis indicates that chromosome 11 contains a number of tumor suppressor genes and that the role of this chromosome in myeloid malignancies is more complex than previously recognized.

## Introduction

Hematological malignancies are broadly categorized into myeloid and lymphoid malignancies, depending on the hematopoietic lineage involved. This study focused on myeloid malignancies, in particular the disease entities acute myeloid leukemia (AML), chronic myeloid leukemia (CML), myelodysplastic syndromes (MDS) as well as the three classical myeloproliferative neoplasms (MPNs) polycythemia vera (PV), essential thrombocythemia (ET) and primary myelofibrosis (PMF). MDS and MPN are in most cases stable, chronic diseases. A fraction of patients, however, develop signs of disease progression such as myelofibrosis or elevated numbers of hematopoietic progenitors in peripheral blood referred to as “accelerated phase”. A transformation to post-MPN or post-MDS AML marks the final stage of the disease and is associated with a very bad prognosis [1]. Genetic aberrations involving chromosome 11 have been widely reported across all hematological malignancies. Translocations of chromosome 11q affecting the 11q23 region have been intensely studied since the late 1970s when the first translocation between chromosomes 11 and 4 was described in acute lymphoblastic leukemia (ALL) [2]. In 1991 the gene that was affected by these translocations on chromosome 11 was identified to be *MLL* (myeloid/lymphoid or mixed-lineage leukemia) [3]. These translocations t(4;11) led to the formation of a fusion gene of *MLL* and *AF4* (ALL1-fused gene from chromosome 4; current official symbol *AFF1*) on chromosome 4 [4]. Since then a variety of translocations involving *MLL* and more than 60 fusion gene partners have been identified. They are found both, in ALL and AML with a high prevalence in infants [5]. In addition to translocations, partial tandem duplications of *MLL* have also been described in AML [6,7]. The internal tandem duplications of *MLL* most often span between exon 3 and exons 9-11 [8], and show a strong association with chromosome 11q trisomies [7]. Classical karyotyping has revealed chromosomal deletions as common genetic changes in chronic lymphoid leukemia (CLL), AML, MDS and other hematological malignancies. A frequently deleted region mapped to 11q23 [9]. In recent years the upcoming of single nucleotide polymorphism (SNP) microarrays has allowed the detection of chromosomal gains and losses at a much higher resolution than with classical cytogenetics. Acquired copy number neutral loss of heterozygosity (LOH) associated with uniparental disomies (UPD), which were previously undetectable by classical cytogenetics, are now recurrently found in hematological malignancies. The first large study in AML using SNP microarrays identified chromosomal aberrations of all three types across the whole genome [10]. We, alongside others, reported such studies in the myeloproliferative neoplasms (MPN) [11-14]. All of these studies observed frequent aberrations on chromosome 11 including gains, losses and UPDs. UPDs were shown to somatically amplify mutant alleles of genes on various chromosomal arms such as 9p (*JAK2*), 1p (*MPL*) or 4q (*TET2*) [15-23]. On the short arm of chromosome 11, mutant alleles of *WT1* were associated with UPDs in AML [24], while *CBL* mutations were associated with UPDs on chromosome 11q in several hematological malignancies[25-27]. *CBL* encodes an E3 ubiquitin ligase that attaches ubiquitin to a number of membrane-associated and cytosolic proteins (such as Flt3, Kit, Jak2 and Mpl) and targets them for degradation [28,29]. In this study, we present a systematic analysis of chromosome 11 in a set of 813 samples across different myeloid malignancies. We used high resolution SNP microarrays and whole exome sequencing to identify novel genetic aberrations of chromosome 11 in myeloid malignancies. We were able to detect commonly aberrant regions on this chromosome and to identify potential target genes of large aberrations. 

## Results and Discussion

### Chromosome 11 aberrations in myeloid malignancies

**Figure 1:**
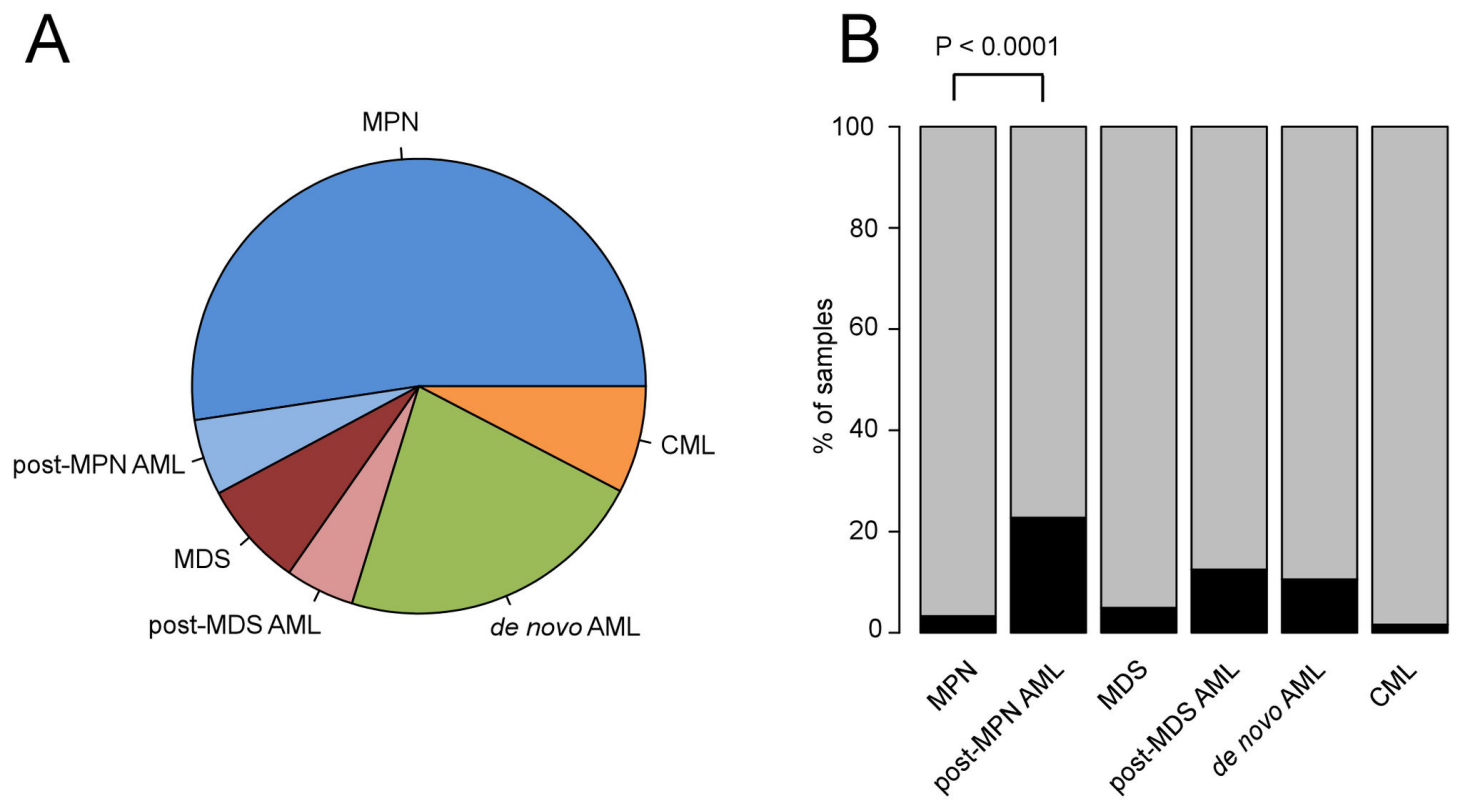
Cohort distribution. **A**: Distribution of the 813 samples analyzed by Affymetrix microarrays according to diagnosis. **B**: Fraction of samples that harbor chromosome 11 aberrations (black bars) for each disease entity in percent. The P-value indicates an association of chromosome 11 aberrations with disease progression in MPN. MPN, myeloproliferative neoplasm; CML, chronic myeloid leukemia; AML, acute myeloid leukemia; MDS, myelodysplastic syndrome.

**Figure 2:**
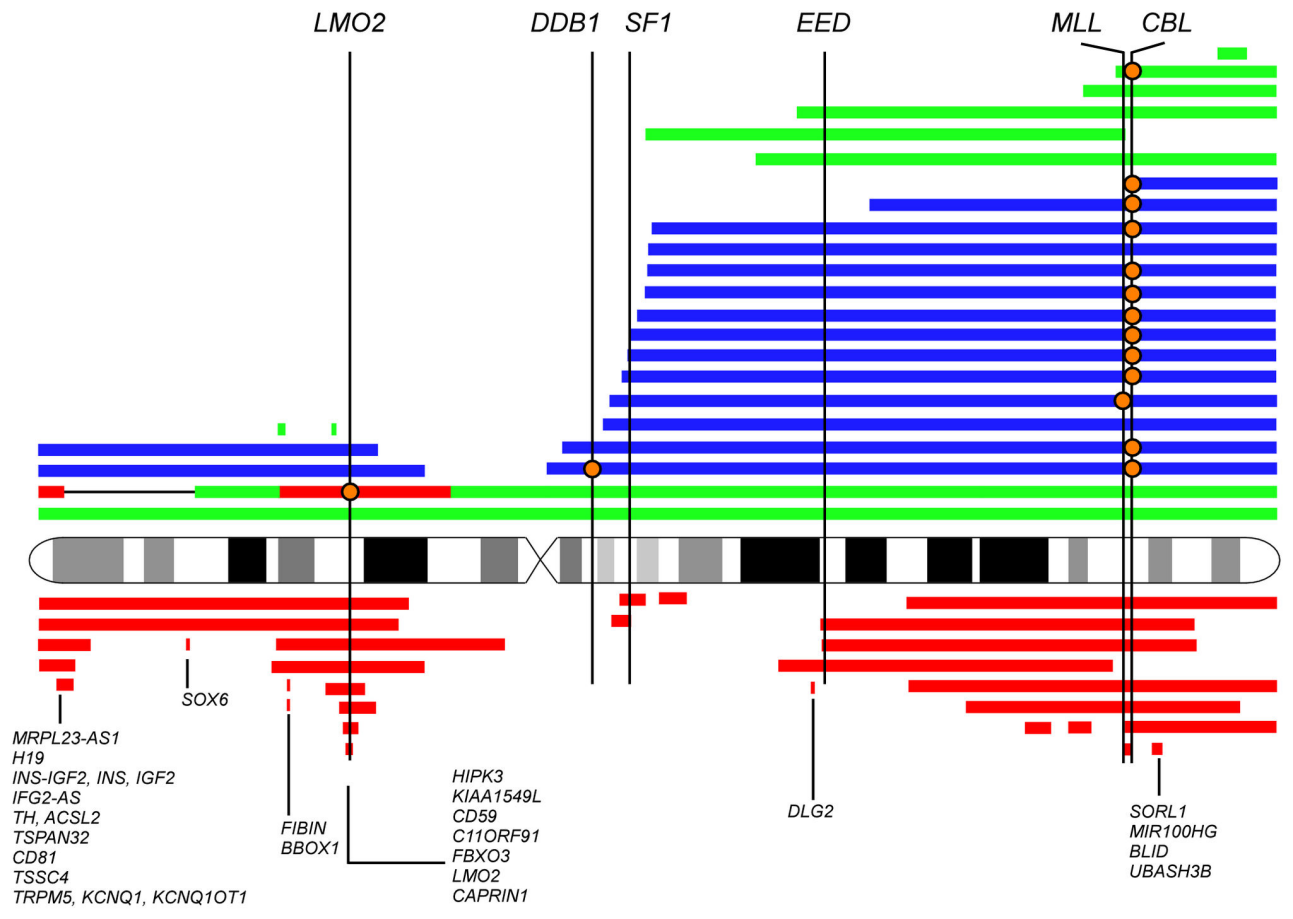
Summary of chromosome 11 aberrations. Large chromosomal aberrations are indicated with colored bars around the ideogram of chromosome 11. Green – gains; red – deletions; blue – uniparental disomies. The position of the bars relative to the chromosome ideogram indicates the position and size of the aberration. For the two patients of whom two samples were analyzed (UPN 23 and UPN 42 – see Table S1) recurrent aberrations are depicted only once. The positions of *CBL, MLL, EED, SF1*, *DDB1* and *LMO2* are indicated by vertical lines. Mutations in these genes are depicted by orange circles along these lines. Common deleted regions are indicated at the bottom of Figure 2 listing the genes they cover. The ideogram depicts G-banding pattern at ~850-band resolution level.

In order to systematically analyze chromosome 11 aberrations in myeloid malignancies, we combined data from a total of 813 blood samples that were genotyped at high-resolution with Affymetrix Genome-Wide Human SNP 6.0 microarrays. This cohort included 180 *de novo* acute myeloid leukemia (AML), 62 chronic myeloid leukemia (CML), 101 myelodysplastic syndrome (MDS), 244 polycythemia vera (PV), 118 essential thrombocythemia (ET) and 108 primary myelofibrosis (PMF) samples (Table 1, Figure 1A). For PV, ET, PMF and MDS, the majority of samples were in chronic phase of the disease, some samples were taken when patients showed signs of disease progression or had transformed to post-chronic phase AML as outlined in Table 1. Chromosome 11 aberrations were detected in 52 of 813 samples (6.4%) (Table 1 and Figure 1B). The 52 samples were from 50 patients, for 2 patients we had 2 samples from different disease stages (Table S1). The samples harbored between 1 to 3 genetic changes on chromosome 11, except for sample 42 which had a complex aberration of chromosome 11 (Table S1). Excluding sample 42, we detected a total of 30 deletions, 11 gains and 17 UPDs (Figure 2 and Table S1). In MPN, aberrations of chromosome 11 significantly associate with post-MPN AML compared to chronic phase MPN (P<0.0001, Fisher’s exact test, Figure 1B). MPN patients that exhibited myelofibrosis or were in the accelerated phase of the disease but had not fully transformed to post-MPN AML (<20% of blasts in peripheral blood or bone marrow) were regarded as chronic phase patients in this analysis. This finding indicates that genes located on chromosome 11 contribute to disease progression if mutated. Associations of chromosome 11q losses of heterozygosity with disease progression or poor prognosis have been described previously in B cell chronic lymphatic leukemia [30] or neuroblastoma [31]. Abnormalities of chromosomal band 11q23 were associated with a poor outcome in infant acute lymphoblastic leukemia (ALL) [32].

**Table 1:** Cohort characteristics.

**Disease entity**	**Specific diagnosis**	**Total (n)**	**With chr 11 lesions (n)**
MPN	Polycythemia vera	177	3
	post-PV MF	48	3
	post-PV AML	19	3
	Essential thrombocythemia	91	2
	post-ET MF	18	1
	post-ET AML	9	1
	Primary Myelofibrosis	85	5
	post-PMF AP	7	0
	post-PMF AML	16	6
MDS	MDS (chronic phase)	61	3
	post-MDS AML	40	5
*de novo* AML	*de novo* AML	180	19
CML	CML	62	1
*total*		*813*	*52*

n, number of samples; MPN, myeloproliferative neoplasms; MDS, myelodysplastic syndrome; AML, acute myeloid leukemia; CML, chronic myeloid leukemia; PV, polycythemia vera; ET, essential thrombocythemia; PMF, primary myelofibrosis; MF, myelofibrosis; AP, accelerated phase; chr, chromosome

### *CBL* is a frequent target of chromosome 11q aberrations

**Figure 3:**
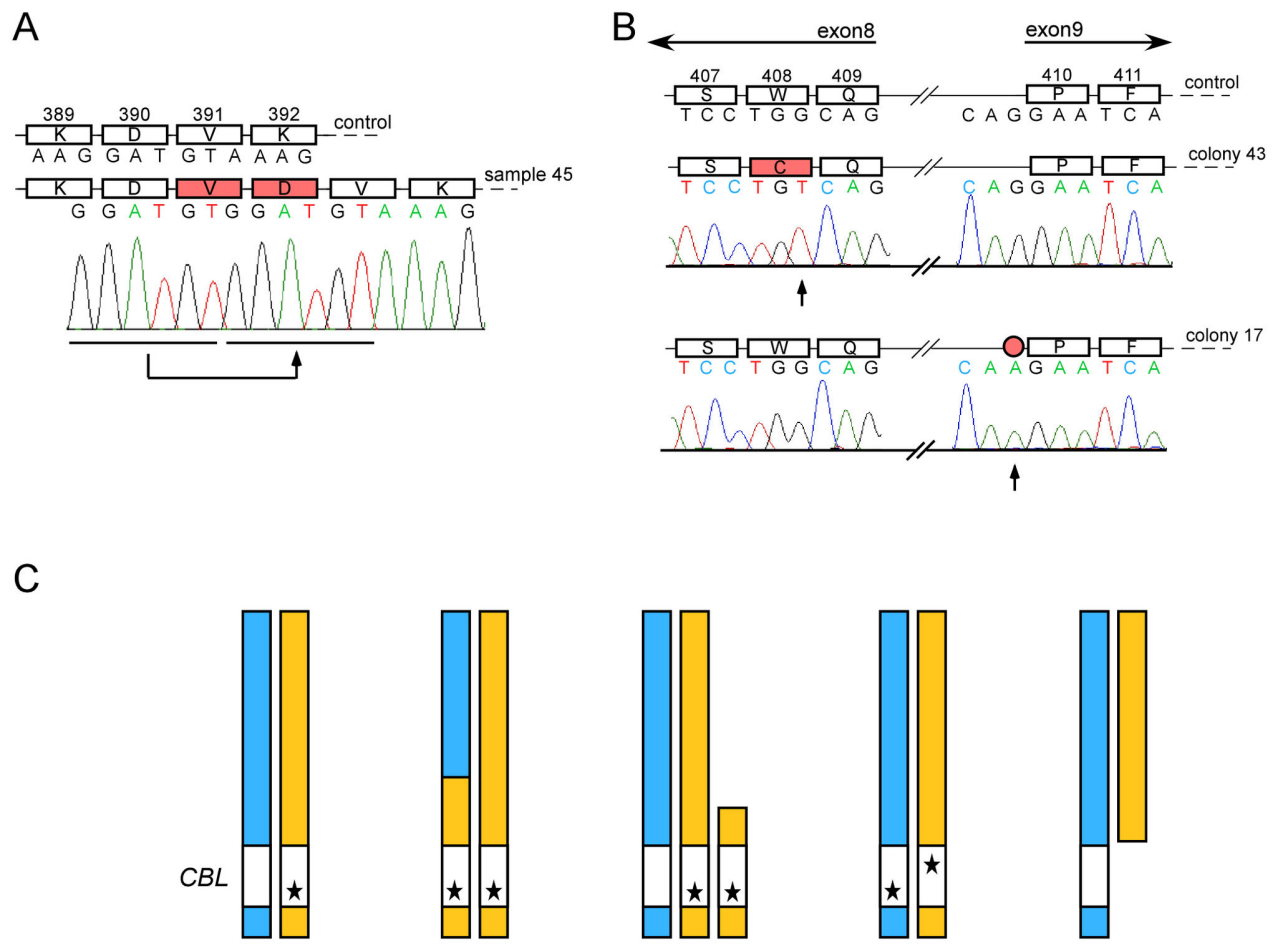
Mutational patterns in *CBL*. **A**: Sample 45 had a 6 bp tandem duplication in CBL leading to the insertion of the amino acids valine (V) and aspartic acid (D) after position 390. **B**: One sample identified in a cohort screen for mutations in *CBL* exons 8 and 9 carried two mutations, one in exon 8 (W408C) and a second one in intron 8 at the splice acceptor site (G to A). PCR subcloning and analysis of colony DNA revealed that the two mutations are on different alleles. Depicted are two representative colonies. Colony 43 has the mutation in exon 8 but not in intron 8 whereas colony 17 shows the opposite case. **A**, **B**: Depicted are the genomic (letters) as well as the respective amino acid (box chains) sequences. Numbers indicate amino acid positions in the Cbl protein. Amino acids, which are substituted due to mutations are in red boxes. The splice site alteration is a red circle. Black arrows indicate the positions of the mutations below the Sanger sequencing traces. **C**: Overview of *CBL* mutagenesis in MPN. Different genetic mechanisms are involved in increasing mutant gene dosage of *CBL*. Each panel shows schematically the two parental copies of chromosomes 11 (blue and yellow) and the *CBL* gene (white rectangles). Mutations are indicated with asterisks. From left to right: heterozygous mutation in *CBL*; uniparental disomy introduces homozygous *CBL* mutations; gain of a part of chromosome 11q leads to a duplication of the *CBL* mutation while one wild type allele is still present; compound heterozygosity established by two different mutations on the different alleles of the *CBL* gene in one cell. In addition, the loss of a part of chromosome 11q deleting one *CBL* allele and leaving the other allele unaffected (wild type *CBL*) is likely to introduce phenotypes due to haploinsufficiency.

We found that UPDs of chromosome 11q are the most recurrent defects in our dataset. A number of studies have shown that 11q UPDs are associated with mutations of the *CBL* gene (ensembl gene ID: ENSG00000110395) [25-27]. Mutations of *CBL* have been described to cluster within exons 8 and 9 or their exon-intron junctions [25-27]. Therefore, we sequenced these two exons of *CBL* in all samples that harbored chromosomal aberrations overlapping the *CBL* locus. Of the 14 patients that had 11q UPDs, we detected SNVs in 9 patients (Table S1). One patient (sample 45) harbored a 6 bp tandem duplication (Figure 3A). Out of 6 patients that had 11q gains overlapping *CBL*, one had a somatic mutation in *CBL* (C384Y in sample 44). PCR subcloning revealed that the gain amplifies the mutant allele (data not shown). No mutations were detected in the 7 patients with deletions overlapping *CBL*. For the patients where we had control tissue available, the somatic origin of the variants detected in *CBL* was confirmed (Table S1). In order to identify mutations in other exons of *CBL* or in other genes that potentially associate with 11q aberrations we performed whole exome sequencing on three samples with 11q uniparental disomies (samples 30, 36 and 50) and two samples with 11q gains (samples 42 and 43) which did not have mutations in exons 8 and 9 of *CBL*. Only one of these samples (36) showed a mutation in *CBL* at the 3’ splice site of exon 7 (Table S1). The variant was somatic and independently validated by Sanger sequencing.

### *CBL* mutations associate with leukemic transformation of MPN

As shown in Figure 1B, we associated chromosome 11 defects with leukemic transformation of MPN. In order to test if *CBL* mutations distinctly associate with disease progression, we sequenced *CBL* exons 8 and 9 in all 44 post-MPN AML samples and 274 chronic phase MPN samples. *CBL* mutations were present in 1.4% of chronic phase and 15.9% of post-MPN leukemic patients, respectively. Thus, *CBL* mutations are significantly associated with post-MPN AML (P=0.0001; Fisher’s exact test). A particularly interesting case in this set of patients had two mutations of *CBL* affecting exon 8 (W408C) and the 5’ splice site of exon 9 (Figure 3B). Both mutations were somatic and PCR subcloning revealed that these two mutations were on independent DNA strands. As all the bacterial clones analyzed contained only one of the mutations and no clones with wild type *CBL* were detected, we concluded that the patient harbors a compound heterozygous progenitor clone with distinct mutations of both *CBL* alleles (Figure 3B).

### Mechanisms that increase mutant *CBL* dosage

Our data on *CBL* suggest that there are several different genetic mechanisms for how the malignant clone can increase the mutant *CBL* allele dosage (Figure 3C). The first mechanism is via mitotic recombination resulting in UPD. The second mechanism is the mutant allele by duplication. Another possibility is the inactivation of wild type alleles by two independent point mutations (compound heterozygosity). Interestingly, it seems possible that loss of a single *CBL* allele (haploinsufficiency) might be oncogenic as 7 patients in our cohort carried hemizygous *CBL* deletions (Figure 2). In support of this hypothesis, heterozygous *Cbl* deficiency in mice showed accelerated blast crisis compared to *Cbl* wild type animals in a BCR-ABL transgenic murine model [27]. In addition, hemizygous deletions of *CBL* have been shown by others in MDS and related disorders [33].

### Mutation of *DDB1* associated with 11q UPD

**Figure 4:**
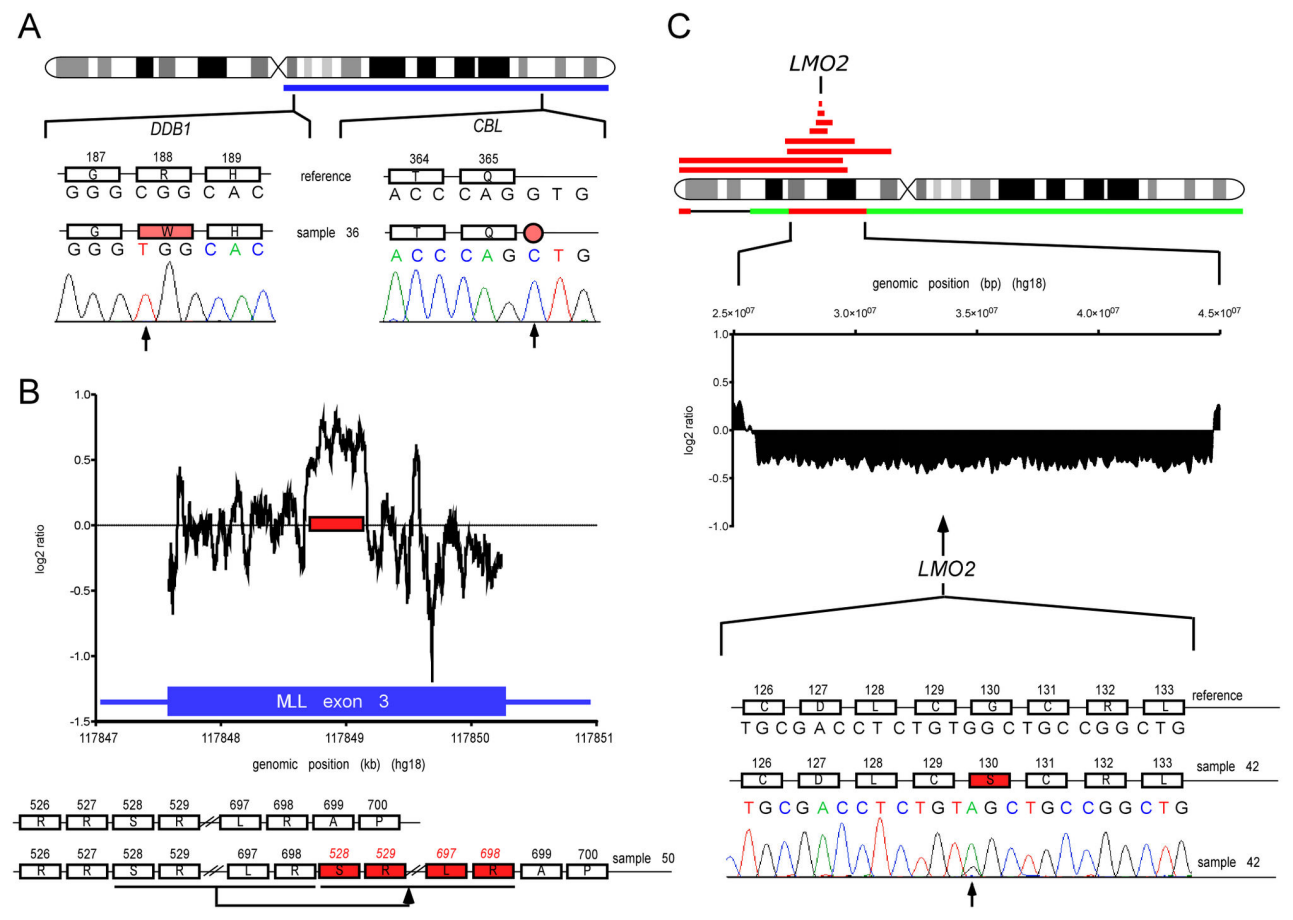
Mutations detected in *DDB1, MLL* and *LMO2*. **A**: Sample 36 harbored an 11q UPD as indicated by the blue bar below the chromosome 11 ideogram. We found two somatic mutations in *DDB1* and *CBL*. As can be seen in the Sanger sequencing traces, both mutations are homozygous due to amplification by the UPD. **B**: In sample 50 a tandem duplication in *MLL* exon 3 was detected. The top graph shows whole exome coverage data across *MLL* exon 3. The data is plotted as the log_2_ ratio of the normalized exome sequencing coverage in the patient sample divided by the median normalized coverage of 8 independent control samples at each genomic position (X-axis). The position of the duplication is indicated by the red bar. Sanger sequencing confirmed an in-frame tandem duplication of 171 amino acids as shown at the bottom. **C**: A common deleted region on chromosome 11p targets *LMO2*. All deletions in the analyzed cohort that span the *LMO2* locus are depicted next to the chromosome 11 ideogram. Red bars indicate deletions, green bars indicate gains. In sample 42, which harbored a deletion spanning the LMO2 locus, we also detected a point mutation in *LMO2*. The middle section shows a signal intensity plot measuring copy number from Affymetrix microarrays. The plot depicts signal intensity (log_2_ scale) differences between the patient and a healthy control pool for each probe (as implemented in the Affymetrix Genotyping Console software). The deletion in sample 42 can be seen as the deviation from 0 for all probes in the deleted genomic region (X-axis). The point mutation in *LMO2* as identified by Sanger sequencing is depicted at the bottom of panel C. **A**, **B** and **C**: Depicted are the genomic (letters) as well as the respective amino acid (box chains) sequences. Numbers above the boxes indicate amino acid positions in the proteins. Amino acids substituted in the patient samples are indicated by red boxes. The red circle indicates a splice site mutation. Reference and mutant sequences are shown. The arrows indicate the site of mutations below the Sanger sequencing traces.

Recently, mutations in the splicing factor 1 gene *SF1* (ensembl gene ID: ENSG00000168066) and a member of the polycomb complex 2 (*EED* – ensembl gene ID: ENSG00000074266) were found in myeloid malignancies [34,35]. Both genes are located on chromosome 11q (Figure 2). We did not find any mutations in these two genes by either whole exome or Sanger sequencing of *EED* and the C-terminal proline-rich region of *SF1* that was found to be the mutational hotspot of the gene [34]. All samples that had aberrations spanning the two loci were analyzed (Figure 2). We performed whole exome sequencing of samples 30, 36, 42, 43 and 50 and attempted to identify genes other than *CBL* that might be associated with aberrations of chromosome 11q (Table S2). In two of the patients (samples 30 and 36), we performed a paired analysis as whole exome sequenced T lymphocyte DNA was available as germline control (samples 30c and 36c). In sample 30 we did not find any somatic mutations with an allelic frequency > 50%, which is expected for variants within the fully clonal 11qUPD region (data not shown). In addition to the somatic mutation in *CBL* described above, sample 36 also harbored another somatic mutation in *DDB1* (ensembl gene ID: ENSG00000167986) (Figure 4A). The *CBL* and *DDB1* mutations in sample 36 were validated by Sanger sequencing and shown to be homozygous and fully clonal (Figure 4A). Both mutations were also detected in an earlier sample of the same patient (sample 23). Sample 23 harbored an 11qUPD in a subclone and accordingly, the mutations in *CBL* and *DDB1* were not fully clonal (data not shown). The *Polyphen2* tool used to predict functional effects of human non-synonymous single nucleotide variants estimated the variant in *DDB1* to be “probably damaging” with the highest probability score of 1. *DDB1* was originally identified in patients suffering from *Xeroderma pigmentosum*, with inherited deficiency in nucleotide excision repair (NER). The gene was cloned together with its binding partner *DDB2*, with which it forms the DDB protein complex [36]. Later, *DDB1* was found to form an E3 ubiquitin ligase complex together with *CUL4A*, *ROC1* and a variable fourth protein that determines the target specificity of the E3 ligase. Overall, more than 30 different proteins have been identified as binding partners [37]. The ubiquitination activity of DDB1-CUL4A-ROC1 complexes has been shown to not only play important roles in NER [38] but also in regulating the expression of the tumor suppressor *CDKN2A* [39]. *CDKN2A* gene expression is associated with histone 3 – lysine 4 (H3K4) trimethylation mediated by the MLL-RBBP5-WDR5 complex. RBBP5 and WDR5 are two of the binding partners of the DDB1-CUL4A-ROC1 complex. *DDB1* expression is required, together with *MLL*, for proper *CDKN2A* transcriptional activation [39]. Thus, inactivating mutations of *DDB1* are likely to contribute to cancer not only by impairing NER, but also by preventing the transcription of tumor suppressor genes. It remains to be seen if the described example of a concerted action of *DDB1* and *MLL* is unique or if there is a systematic relationship between these two genes that might play a role in hematologic malignancies.

In the remaining three samples that were whole exome sequenced (samples 42, 43 and 50) we identified a number of SNVs and small indels that we could validate by Sanger sequencing (Table S3). Only one gene appeared to be recurrent in this dataset, *HEPHL1*. Two patients (samples 42 and 50) harbored both an SNV in the *HEPHL1* gene as indicated in Table S3. The function of *HEPHL1* is not known. As we did not have control tissue available from these patients, we were unable to identify the somatic or germline origin of these variants.

### Tandem duplication in exon 3 of *MLL* associated with 11q UPD

In order to find small scale genetic alterations that are either too small to be detected by Affymetrix microarrays or too large to be detected by standard exome sequencing pipelines, we analyzed exome coverage data that we gained after alignment of the short sequence reads to the human reference genome. We compared the coverage data of each of the five exome datasets to a set of control samples to identify regions of focal deletions or gains on chromosome 11q. In sample 50, we were able to detect a focal amplification in exon 3 of *MLL* (ensembl gene ID: ENSG00000118058) (Figure 4B). Independent analysis by Sanger sequencing revealed a 513 bp tandem duplication in *MLL* exon 3. This duplication translates to an in-frame duplication of 171 amino acids from position 528 to 698 of the MLL protein (uniprot ID Q03164-1) (Figure 4B). We did not have control tissue of this patient available to confirm the somatic origin of this duplication. However, the duplication was not present in 196 control subjects ruling out the possibility of a common germline polymorphism. Tandem duplications in *MLL* have been described but usually affect the region from exon 3 to exon 9, 10 or 11 [8]. Small tandem duplications such as the 513 bp within exon 3 detected in our study have not been reported so far. 

### Chromosome 11p defects associate with de novo AML or target *LMO2*

On chromosome 11p, we identified a total of 4 CDRs (Figure 2). The most telomeric CDR contained 14 genes. Interestingly, we found a significant association of aberrations spanning this CDR with de novo AML compared to secondary AML (P = 0.013). It is likely that one or more of the genes in this region play a particular role in *de novo* AML pathogenesis. The most centromeric CDR on the short arm of chromosome 11, defined by a deletion in sample 39 contains the *LMO2* gene (ensembl gene ID: ENSG00000135363). In sample 32, where we detected a deletion spanning the *LMO2* locus (Table S1), we also found an SNV in *LMO2* in the remaining allele (c.G388A; p. G130S, Uniprot ID P25791-3) that was hemizygous in Sanger sequencing traces (Figure 4C). The *Polyphen2* tool estimated the variant to be “probably damaging” with the highest probability score of 1. Due to lack of control tissue in this patient we could not analyze the somatic or germline origin of this SNV. Based on the available data we postulate that there is a full loss of *LMO2* activity in this patient. We tested all other patients with aberrations overlapping the *LMO2* locus, but were unable to find any mutations in the coding region or at splice sites of *LMO2* (data not shown). The deletions were detected across several different pathologies. *LMO2* is frequently involved in translocations in T-cell leukemia [40]. It is expressed in different fetal tissues [41] and the full knockout in the mouse is known to be embryonic lethal [42]. Warren et al. showed that *LMO2* is essential for erythroid development in the mouse. Deficiency in erythropoiesis was detected at E9.75. They confirmed by in vitro differentiation assays that this defect is intrinsic to the hematopoietic system and specific for the erythroid lineage [42]. Interestingly the patient in our study showed anemia with an hemoglobin level of 97 g/L at the time of sampling. 

### Concluding remarks and perspectives

In this study we applied a chromosome centered genetic analysis of myeloid malignancies. The rationale of this approach is that those chromosomes that exhibit frequent chromosomal defects might also harbor point mutations in the target genes of deletions, gains or UPD. Combining SNP microarray analysis and exome sequencing may increase the likelihood of identification of novel tumor suppressor genes or oncogenes. Applying this approach we systematically analyzed chromosome 11 in myeloid malignancies and detected a large complexity of genetic aberrations especially in patients with AML (*de novo* or secondary to MPN and MDS). The various genetic lesions of chromosome 11 in myeloid malignancies target *CBL, MLL, DDB1, LMO2* and possibly other tumor suppressor genes that we could not identify in this study. The marked cytogenetic complexity associated with AML points towards a highly individual course of disease progression in each patient and might explain the current difficulty in treating patients that have transformed to AML. 

Our data indicates that genetic stratification of patients into comparable groups at advanced disease stage will be extremely challenging or impossible due to highly individual mutagenesis profiles. Despite individual mutagenesis profiles, it is possible that common molecular features may emerge (based on gene expression and/or protein phosphorylation profiles). Systems level approaches may help in overcoming this obstacle of genetic heterogeneity, opening up the possibility of targeted therapies in the future. Based on current knowledge, treatment efforts in the chronic phase of myeloid malignancies should not only focus on correction of blood counts but also focus on prevention of disease progression as therapeutic intervention in advance disease stages are predicted to be difficult as the genetic complexity of tumors reach an immense scale. 

## Materials and Methods

### Ethics statement

Peripheral blood samples were collected from patients after written informed consent. Sample collection was approved by local ethics committees. These were the “Ethik Kommission der Medizinischen Universität Wien” for samples collected in Austria, the “Comitato di Bioetica” for samples collected at the Fondazione Istituto di Ricovero e Cura a Carattere Scientifico (IRCCS) Policlinico San Matteo, Pavia, Italy, the “Local Ethical Committee of Azienda Ospedaliera-Universitaria Careggi, Firenze” for samples collected at the University of Florence, Italy, the Ethics Committee of University Hospital Brno for samples collected at the Masaryk University Brno, Czech Republic, and the ”Eticki odbor Klinickog centra Srbije” for samples collected at the University of Belgrade, Serbia.

### Patient samples

We analyzed a total of 813 samples from 773 patients. For 40 patients we had two samples available, which were in all cases from two different disease stages. Detailed information on the studied cohort is provided in Table 1. Genomic DNA was isolated from whole blood, granulocytes or mononuclear cell fractions according to standard procedures. For a subset of patients we had control tissue DNA available, extracted from either buccal mucosa cells, T lymphocyte fractions of peripheral blood or cultured skin fibroblasts.

### Microarray analysis and whole exome sequencing

The genomic DNA was processed and hybridized to Genome-Wide Human SNP 6.0 arrays (Affymetrix, Santa Clara, CA) according to the manufacturer’s instructions. Chromosomal copy number changes and UPDs were detected using the Genotyping Console version 3.0.2 software (Affymetrix). 

Five tumor samples (30, 36, 42, 43 and 50) and two matched control samples (30c and 36c) were analyzed by whole exome sequencing (Table S1). Genomic DNA libraries were generated either by using the NEBNext DNA Sample Prep Reagent Set 1 (New England Biolabs, Ipswich, MA) for sample 30 or the TruSeq DNA Sample Prep-Kit v2 (Illumina, San Diego, CA) for samples 36, 42, 43, 50, 30c and 36c. Whole exome enrichment was performed using the Sure Select Human All Exon Kit (Agilent, Santa Clara, CA) for sample 30 or the TruSeq Exome Enrichment Kit (Illumina) for the six other samples. The exome - enriched libraries were hybridized to Illumina flowcells V1 (sample 30) or V3 (other samples) and sequenced using the Illumina HiSeq 2000 instrument. A summary of all samples and sequencing parameters is provided in Table S2. The sequence reads were aligned against the human reference genome (hg18) using BWA v0.5.9 [43]. Subsequently, the aligned samples were post processed using GATK v1.5 [44] following their best practices guidelines (v3). Briefly, this comprises marking PCR-duplicate reads, recalibrating the base quality scores and local realignment around insertions/deletions (indels). Variant discovery was performed on the post-processed alignment files using GATK’s Unified Genotyper [45]. The final variant lists were generated using GATK’s Variant Quality Score Recalibrator using the suggested filtering parameters.

For samples 30 and 36 where control tissue DNA was whole exome sequenced (samples 30c and 36c) we performed an analysis for somatic mutations by using the *VarScan2* software with default parameters[46] starting from the post – processed alignment files generated by GATK. 

For samples 42, 43 and 50 the final variant lists of the GATK Unified Genotyper were filtered for single nucleotide variants (SNVs) and indels on chromosome 11 that were passing filter criteria according to the GATK best practice guidelines v3 and that were not annotated in dbSNP137. Gene annotation was done using the *ANNOVAR* tool version 2012-02-23 [47].

The raw data of microarray analysis and whole exome sequencing are deposited in the ArrayExpress database under the accession numbers E-MTAB-1845 and E-MTAB-1850, respectively. 

### Coverage analysis from whole exome sequencing data

The analysis was performed for the five tumor samples, which had been whole exome sequenced. *Samtools 0.1.18* [48] was used with the “depth” option to retrieve coverage data for chromosome 11 from the post – processed alignment files generated by the GATK analysis pipeline. The coverage for each base on chromosome 11 in a particular patient was normalized by the summarized coverage of all bases of chromosome 11 in that particular patient. The normalized coverage of sample 30 was compared to the median normalized coverage of a set of 5 independent control samples that had been processed and whole exome sequenced with similar chemistry and instrumentation as sample 30. A similar adequate control set of 8 independent control samples was generated for samples 36, 42, 43 and 50. All of the control samples used showed wild-type chromosome 11 as analyzed by Genome-Wide Human SNP 6.0 arrays (Affymetrix, data not shown). 

### PCR, Sanger sequencing, PCR subcloning

Primers for PCR were designed using the *Primer 3* tool (http://www.bioinformatics.nl/cgi-bin/primer3plus/primer3plus.cgi) or the *ExonPrimer* tool (http://ihg.gsf.de/ihg/ExonPrimer.html) except for the primers amplifying *CBL* exons 8 and 9 which were taken from a publication by Sanada et al [27]. Primer sequences and PCR conditions are listed in Table S4. PCRs were performed using the AmpliTaq Gold DNA Polymerase with Gold Buffer and MgCl2 solution (Applied Biosystems / Life Technologies, Paisley, UK) or the AmpliTaq Gold 360 Mastermix (Applied Biosystems). Sanger sequencing was performed using the BigDye Terminator v3.1 Cycle Sequencing kit and the 3130xl Genomic Analyzer (Applied Biosystems). Sequence analysis was done using the Sequencher Software 4.9 (Gene Codes, Ann Arbor, MI). For PCR product subcloning the TOPO Cloning Kit (Invitrogen / Life Technologies, Paisley, UK) was used according to manufacturer’s instructions. PCR products derived from single bacterial clones were sequenced as described above.

### Statistical analysis and plots

Fisher’s exact tests were performed using Graphpad QuickCalcs (www.graphpad.com/quickcalcs). The plots depicting cohort distributions in Figure 1 were done using R version 2.8.1 (2008-12-22) [49]. The coverage plot in Figure 4B and the signal intensity plot in Figure 4C were done using GraphPad Prism version 5.0d for Mac OS X, GraphPad Software (San Diego, CA), www.graphpad.com.

## Supporting Information

Table S1 **List of genetic aberrations on chromosome 11 detected in 52 samples with myeloid malignancies.**
(XLS)

Table S2 **Whole exome sequencing parameters.**
(XLS)

Table S3 **Validated whole exome sequencing results overlapping chromosome 11q aberrations.**
(XLS)

Table S4 **PCR primers and program.**
(XLS)

## References

[1] Sverdlow S, Campl E, Harris N, Jaffe E, Pileri S et al. (2008) WHO Classification of Tumours of Haematopoietic and Lymphoid Tissues. Lyon: International Agency for Research on Cancer.

[2] Oshimura M, Freeman AI, Sandberg AA (1977) Chromosomes and causation of human cancer and leukemia. XXVI. Binding studies in acute lymphoblastic leukemia (ALL). Cancer 40: 1161-1172. doi:10.1002/1097-0142(197709)40:3. PubMed: 268996. 268996

[3] Ziemin-van der Poel S, McCabe NR, Gill HJ, Espinosa R 3rd, Patel Y et al. (1991) Identification of a gene, MLL, that spans the breakpoint in 11q23 translocations associated with human leukemias. Proc Natl Acad Sci U S A 88: 10735-10739. doi:10.1073/pnas.88.23.10735. PubMed: 1720549. 1720549 PMC53005

[4] Gu Y, Nakamura T, Alder H, Prasad R, Canaani O et al. (1992) The t(4;11) chromosome translocation of human acute leukemias fuses the ALL-1 gene, related to Drosophila trithorax, to the AF-4 gene. Cell 71: 701-708. doi:10.1016/0092-8674(92)90603-A. PubMed: 1423625. 1423625

[5] Liu H, Cheng EH, Hsieh JJ (2009) MLL fusions: pathways to leukemia. Cancer Biol Ther 8: 1204-1211. doi:10.4161/cbt.8.13.8924. PubMed: 19729989. 19729989 PMC3289713

[6] Schichman SA, Caligiuri MA, Gu Y, Strout MP, Canaani E et al. (1994) ALL-1 partial duplication in acute leukemia. Proc Natl Acad Sci U S A 91: 6236-6239. doi:10.1073/pnas.91.13.6236. PubMed: 8016145. 8016145 PMC44173

[7] Caligiuri MA, Strout MP, Schichman SA, Mrózek K, Arthur DC et al. (1996) Partial tandem duplication of ALL1 as a recurrent molecular defect in acute myeloid leukemia with trisomy 11. Cancer Res 56: 1418-1425. PubMed: 8640834. 8640834

[8] Schnittger S, Kinkelin U, Schoch C, Heinecke A, Haase D et al. (2000) Screening for MLL tandem duplication in 387 unselected patients with AML identify a prognostically unfavorable subset of AML. Leukemia 14: 796-804. doi:10.1038/sj.leu.2401773. PubMed: 10803509. 10803509

[9] Monni O, Knuutila S (2001) 11q deletions in hematological malignancies. Leuk Lymphoma 40: 259-266. doi:10.3109/10428190109057924. PubMed: 11426547. 11426547

[10] Gupta M, Raghavan M, Gale RE, Chelala C, Allen C et al. (2008) Novel regions of acquired uniparental disomy discovered in acute myeloid leukemia. Genes Chromosomes Cancer 47: 729-739. doi:10.1002/gcc.20573. PubMed: 18506749. 18506749

[11] Thoennissen NH, Krug UO, Lee DH, Kawamata N, Iwanski GB et al. (2010) Prevalence and prognostic impact of allelic imbalances associated with leukemic transformation of Philadelphia chromosome-negative myeloproliferative neoplasms. Blood 115: 2882-2890. doi:10.1182/blood-2009-07-235119. PubMed: 20068225. 20068225 PMC2854432

[12] Stegelmann F, Bullinger L, Griesshammer M, Holzmann K, Habdank M et al. (2010) High-resolution single-nucleotide polymorphism array-profiling in myeloproliferative neoplasms identifies novel genomic aberrations. Haematologica 95: 666-669. doi:10.3324/haematol.2009.013623. PubMed: 20015882. 20015882 PMC2857198

[13] Klampfl T, Harutyunyan A, Berg T, Gisslinger B, Schalling M et al. (2011) Genome integrity of myeloproliferative neoplasms in chronic phase and during disease progression. Blood 118: 167-176. doi:10.1182/blood-2011-01-331678. PubMed: 21531982. 21531982

[14] Rumi E, Harutyunyan A, Elena C, Pietra D, Klampfl T et al. (2011) Identification of genomic aberrations associated with disease transformation by means of high-resolution SNP array analysis in patients with myeloproliferative neoplasm. Am J Hematol 86: 974-979. doi:10.1002/ajh.22166. PubMed: 21953568. 21953568

[15] Kralovics R, Passamonti F, Buser AS, Teo SS, Tiedt R et al. (2005) A gain-of-function mutation of JAK2 in myeloproliferative disorders. N Engl J Med 352: 1779-1790. doi:10.1056/NEJMoa051113. PubMed: 15858187. 15858187

[16] Levine RL, Wadleigh M, Cools J, Ebert BL, Wernig G et al. (2005) Activating mutation in the tyrosine kinase JAK2 in polycythemia vera, essential thrombocythemia, and myeloid metaplasia with myelofibrosis. Cancer Cell 7: 387-397. doi:10.1016/j.ccr.2005.03.023. PubMed: 15837627. 15837627

[17] James C, Ugo V, Le Couédic JP, Staerk J, Delhommeau F et al. (2005) A unique clonal JAK2 mutation leading to constitutive signalling causes polycythaemia vera. Nature 434: 1144-1148. doi:10.1038/nature03546. PubMed: 15793561. 15793561

[18] Baxter EJ, Scott LM, Campbell PJ, East C, Fourouclas N et al. (2005) Acquired mutation of the tyrosine kinase JAK2 in human myeloproliferative disorders. Lancet 365: 1054-1061. doi:10.1016/S0140-6736(05)71142-9. PubMed: 15781101. 15781101

[19] Pardanani AD, Levine RL, Lasho T, Pikman Y, Mesa RA et al. (2006) MPL515 mutations in myeloproliferative and other myeloid disorders: a study of 1182 patients. Blood 108: 3472-3476. doi:10.1182/blood-2006-04-018879. PubMed: 16868251. 16868251

[20] Szpurka H, Gondek LP, Mohan SR, Hsi ED, Theil KS et al. (2009) UPD1p indicates the presence of MPL W515L mutation in RARS-T, a mechanism analogous to UPD9p and JAK2 V617F mutation. Leukemia 23: 610-614. doi:10.1038/leu.2008.249. PubMed: 18818701. 18818701

[21] Buxhofer-Ausch V, Gisslinger H, Berg T, Gisslinger B, Kralovics R (2009) Acquired resistance to interferon alpha therapy associated with homozygous MPL-W515L mutation and chromosome 20q deletion in primary myelofibrosis. Eur J Haematol 82: 161-163. doi:10.1111/j.1600-0609.2008.01183.x. PubMed: 19018861. 19018861

[22] Delhommeau F, Dupont S, Della Valle V, James C, Trannoy S et al. (2009) Mutation in TET2 in myeloid cancers. N Engl J Med 360: 2289-2301. doi:10.1056/NEJMoa0810069. PubMed: 19474426. 19474426

[23] Tefferi A, Pardanani A, Lim KH, Abdel-Wahab O, Lasho TL et al. (2009) TET2 mutations and their clinical correlates in polycythemia vera, essential thrombocythemia and myelofibrosis. Leukemia 23: 905-911. doi:10.1038/leu.2009.47. PubMed: 19262601. 19262601 PMC4654629

[24] Fitzgibbon J, Smith LL, Raghavan M, Smith ML, Debernardi S et al. (2005) Association between acquired uniparental disomy and homozygous gene mutation in acute myeloid leukemias. Cancer Res 65: 9152-9154. doi:10.1158/0008-5472.CAN-05-2017. PubMed: 16230371. 16230371

[25] Dunbar AJ, Gondek LP, O'Keefe CL, Makishima H, Rataul MS et al. (2008) 250K single nucleotide polymorphism array karyotyping identifies acquired uniparental disomy and homozygous mutations, including novel missense substitutions of c-Cbl, in myeloid malignancies. Cancer Res 68: 10349-10357. doi:10.1158/0008-5472.CAN-08-2754. PubMed: 19074904. 19074904 PMC2668538

[26] Grand FH, Hidalgo-Curtis CE, Ernst T, Zoi K, Zoi C et al. (2009) Frequent CBL mutations associated with 11q acquired uniparental disomy in myeloproliferative neoplasms. Blood 113: 6182-6192. doi:10.1182/blood-2008-12-194548. PubMed: 19387008. 19387008

[27] Sanada M, Suzuki T, Shih LY, Otsu M, Kato M et al. (2009) Gain-of-function of mutated C-CBL tumour suppressor in myeloid neoplasms. Nature 460: 904-908. doi:10.1038/nature08240. PubMed: 19620960. 19620960

[28] Thien CB, Langdon WY (2001) Cbl: many adaptations to regulate protein tyrosine kinases. Nat Rev Mol Cell Biol 2: 294-307. doi:10.1038/35067100. PubMed: 11283727. 11283727

[29] Schmidt MH, Dikic I (2005) The Cbl interactome and its functions. Nat Rev Mol Cell Biol 6: 907-918. doi:10.1038/nrm1762. PubMed: 16227975. 16227975

[30] Döhner H, Stilgenbauer S, James MR, Benner A, Weilguni T et al. (1997) 11q deletions identify a new subset of B-cell chronic lymphocytic leukemia characterized by extensive nodal involvement and inferior prognosis. Blood 89: 2516-2522. PubMed: 9116297. 9116297

[31] Luttikhuis ME, Powell JE, Rees SA, Genus T, Chughtai S et al. (2001) Neuroblastomas with chromosome 11q loss and single copy MYCN comprise a biologically distinct group of tumours with adverse prognosis. Br J Cancer 85: 531-537. doi:10.1054/bjoc.2001.1960. PubMed: 11506492. 11506492 PMC2364087

[32] Chen CS, Sorensen PH, Domer PH, Reaman GH, Korsmeyer SJ et al. (1993) Molecular rearrangements on chromosome 11q23 predominate in infant acute lymphoblastic leukemia and are associated with specific biologic variables and poor outcome. Blood 81: 2386-2393. PubMed: 8481519. 8481519

[33] Makishima H, Cazzolli H, Szpurka H, Dunbar A, Tiu R et al. (2009) Mutations of e3 ubiquitin ligase cbl family members constitute a novel common pathogenic lesion in myeloid malignancies. J Clin Oncol 27: 6109-6116. doi:10.1200/JCO.2009.23.7503. PubMed: 19901108. 19901108 PMC3040009

[34] Yoshida K, Sanada M, Shiraishi Y, Nowak D, Nagata Y et al. (2011) Frequent pathway mutations of splicing machinery in myelodysplasia. Nature 478: 64-69. doi:10.1038/nature10496. PubMed: 21909114. 21909114

[35] Score J, Hidalgo-Curtis C, Jones AV, Winkelmann N, Skinner A et al. (2012) Inactivation of polycomb repressive complex 2 components in myeloproliferative and myelodysplastic/myeloproliferative neoplasms. Blood 119: 1208-1213. doi:10.1182/blood-2011-07-367243. PubMed: 22053108. 22053108

[36] Dualan R, Brody T, Keeney S, Nichols AF, Admon A et al. (1995) Chromosomal localization and cDNA cloning of the genes (DDB1 and DDB2) for the p127 and p48 subunits of a human damage-specific DNA binding protein. Genomics 29: 62-69. doi:10.1006/geno.1995.1215. PubMed: 8530102. 8530102

[37] Angers S, Li T, Yi X, MacCoss MJ, Moon RT et al. (2006) Molecular architecture and assembly of the DDB1-CUL4A ubiquitin ligase machinery. Nature 443: 590-593. PubMed: 16964240. 16964240 10.1038/nature05175

[38] Iovine B, Iannella ML, Bevilacqua MA (2011) Damage-specific DNA binding protein 1 (DDB1): a protein with a wide range of functions. Int J Biochem Cell Biol 43: 1664-1667. doi:10.1016/j.biocel.2011.09.001. PubMed: 21959250. 21959250

[39] Kotake Y, Zeng Y, Xiong Y (2009) DDB1-CUL4 and MLL1 mediate oncogene-induced p16INK4a activation. Cancer Res 69: 1809-1814. doi:10.1158/0008-5472.CAN-08-2739. PubMed: 19208841. 19208841 PMC2653104

[40] Royer-Pokora B, Loos U, Ludwig WD (1991) TTG-2, a new gene encoding a cysteine-rich protein with the LIM motif, is overexpressed in acute T-cell leukaemia with the t(11;14)(p13;q11). Oncogene 6: 1887-1893. 1923511

[41] Foroni L, Boehm T, White L, Forster A, Sherrington P et al. (1992) The rhombotin gene family encode related LIM-domain proteins whose differing expression suggests multiple roles in mouse development. J Mol Biol 226: 747-761. doi:10.1016/0022-2836(92)90630-3. PubMed: 1507224. 1507224

[42] Warren AJ, Colledge WH, Carlton MB, Evans MJ, Smith AJ et al. (1994) The oncogenic cysteine-rich LIM domain protein rbtn2 is essential for erythroid development. Cell 78: 45-57. doi:10.1016/0092-8674(94)90571-1. PubMed: 8033210. 8033210

[43] Li H, Durbin R (2009) Fast and accurate short read alignment with Burrows-Wheeler transform. Bioinformatics 25: 1754-1760. doi:10.1093/bioinformatics/btp324. PubMed: 19451168. 19451168 PMC2705234

[44] McKenna A, Hanna M, Banks E, Sivachenko A, Cibulskis K et al. (2010) The Genome Analysis Toolkit: a MapReduce framework for analyzing next-generation DNA sequencing data. Genome Res 20: 1297-1303. doi:10.1101/gr.107524.110. PubMed: 20644199. 20644199 PMC2928508

[45] DePristo MA, Banks E, Poplin R, Garimella KV, Maguire JR et al. (2011) A framework for variation discovery and genotyping using next-generation DNA sequencing data. Nat Genet 43: 491-498. doi:10.1038/ng.806. PubMed: 21478889. 21478889 PMC3083463

[46] Koboldt DC, Zhang Q, Larson DE, Shen D, McLellan MD et al. (2012) VarScan 2: somatic mutation and copy number alteration discovery in cancer by exome sequencing. Genome Res 22: 568-576. doi:10.1101/gr.129684.111. PubMed: 22300766. 22300766 PMC3290792

[47] Wang K, Li M, Hakonarson H (2010) ANNOVAR: functional annotation of genetic variants from high-throughput sequencing data. Nucleic Acids Res 38: e164. doi:10.1093/nar/gkq603. PubMed: 20601685. 20601685 PMC2938201

[48] Li H, Handsaker B, Wysoker A, Fennell T, Ruan J et al. (2009) The Sequence Alignment/Map format and SAMtools. Bioinformatics 25: 2078-2079. doi:10.1093/bioinformatics/btp352. PubMed: 19505943. 19505943 PMC2723002

[49] R Development Core Team (2008) R: A language and environment for statistical. R Foundation for Statistical Computing, Vienna, Austria ISBN 3-900051-07-0 Available: http://www.R-project.org.

